# Treatment of Heartburn: A Survey of Ontario and Québec Community Pharmacists

**DOI:** 10.3390/pharmacy12030081

**Published:** 2024-05-22

**Authors:** Nardine R. Nakhla, Sherilyn K. D. Houle, Jeffrey G. Taylor

**Affiliations:** 1 School of Pharmacy, University of Waterloo, Waterloo, ON N2L 3G1, Canada; sherilyn.houle@uwaterloo.ca; 2 College of Pharmacy and Nutrition, University of Saskatchewan, Saskatoon, SK S7N 5E5, Canada

**Keywords:** drug scheduling, self-care, pharmacists, community pharmacy services, heartburn, GERD, nonprescription drugs, self-medication

## Abstract

The shift of proton pump inhibitors (PPIs) from prescription to nonprescription (nonRx) status in Canada has altered pharmacist treatment options for heartburn. This report examines pharmacist approaches to therapy based on case severity; pharmacist confidence and consult duration were also explored. A 2022 online survey gathered data from Ontario and Québec pharmacists regarding their therapeutic approaches for two hypothetical heartburn cases. A total of 715 pharmacists participated, with most having 1–10 years of experience. In Ontario, common choices for the milder case included a solo histamine-2 receptor antagonist (H2RA) (21.2%), combination H2RA + antacid (29.4%), and nonRx PPI (22.3%). For the more severe case, common choices for Québec were switches to nonRx H2RA (22.1%), combination H2RA + antacid (13.4%), a nonRx PPI (24.9%), or prescription PPI (22.5%). Pharmacists often recommended switching medications or referring patients with recurring symptoms after seven days. The approaches varied significantly between cases and provinces. The Ontario pharmacists favoured a combination H2RA + antacid for the milder case, while the Québec pharmacists preferred a solo H2RA. For the more severe case, both groups often chose nonRx H2RA followed by nonRx PPI. Despite the differences, the pharmacists demonstrated confidence in managing these situations. These findings highlight potential debates regarding optimal therapeutic approaches and the impact of drug scheduling on patient care.

## 1. Introduction

Minor ailments are a common occurrence. In very early work using symptom diaries, tiredness, headache, backache, and colds ranked as the second, third, fourth, and sixth most common experiences over a one-month period [1]. More recently, in a survey of almost 2500 UK citizens, feeling tired, difficulty sleeping, sore throat, coughs and colds, headaches, joint pain, and heartburn ranked very high in terms of frequency across a spectrum of 25 possible symptoms [2]. Of course, it can be debated as to whether all symptoms listed were necessarily ‘minor’ situations.

In Canada specifically, adults experience an estimated 82 million episodes of headaches/migraines, 85 million episodes of colds/flu, and 46 million episodes of indigestion/heartburn over the course of one year [3].

Turning our attention specifically to heartburn, five million people suffer from it and/or acid regurgitation at least once each week, which results in tremendous expense in terms of lost productivity [4]. In one Canadian city, remedies for upset stomach ranked fourth in frequency of product use in a survey of adults, with 27.1 percent of the respondents saying they had used one within a previous six-month period [5].

Some cases will eventually enter the formal healthcare system with a visit to a doctor [6], with dyspepsia ranking as the fourteenth most common condition seen by primary care physicians in 12 countries.

Several reports have noted the benefits that pharmacist involvement can have on patient care for heartburn [7,8,9,10,11]. In Swedish pharmacies (where over-the-counter (OTC) medicine counselling was tracked), dyspepsia was the third most common situation (7.2 percent) [12], where the majority of cases that required medical referral were in fact referred to such care [13], but where only one patient in five advised to see a physician actually did so [14]. In Australia, pharmacists responded to a disguised shopper with NSAID-induced reflux [15]. Across 223 pharmacy visits, recommendations for treatment included a histamine-2 receptor antagonist (H2RA) (57 percent), an antacid (19 percent), and a proton pump inhibitor (PPI) (18 percent). A referral to a doctor was made in 63 percent of visits.

The treatment options available in Canada for the relief of heartburn and acid reflux are similar and include antacids, antacid-alginates, H2RAs, and PPIs. When used for mild, infrequent symptoms and for no more than 2 weeks at lower dosages, these agents are generally available without a prescription. If symptoms are more moderate to severe in nature, recur or worsen, and for treatment durations longer than 2 weeks, prescription products with higher doses are usually used. For example, esomeprazole 20 mg once daily for 14 days is a standard nonRx PPI dosage, whereas 40 mg once daily for a longer duration (4 to 8 weeks) is a standard prescription dosage [16]. While the role of Canadian pharmacists has been discussed from a clinical standpoint [16,17], how they deal with heartburn at the community level has not been well described. Renaud and Glavas, however, did report on a commercial venture that was designed to help pharmacists manage patients with dyspepsia [18]. Twenty pharmacies in the Windsor, ON area recruited 500 patients over three months. Of those, 53 percent were referred to their physician. With respect to therapy decisions (circa 2001), 67 were switched to a PPI, 15 had a dosage change, and 26 were given additional therapy to what was then being used.

Current pharmacist behavior is of interest, given that prior to the first de-scheduling (movement from prescription to nonRx status) of a PPI (omeprazole) in Canada, pharmacists at the time (2007–2009) were not supportive of that possibility, with pharmacists citing the complexity of the disease and drug management as well as the need for additional training as barriers [19]. It is also quite likely that there were provincial differences in how pharmacists responded to the issues raised.

This research aimed to determine current community pharmacist practice in the treatment of heartburn considering drug class and prescription or nonRx status, as well as confidence in care provision and the estimated duration per consultation across cases of differing complexity. In a companion article (awaiting publication) to the report described here, Ontario pharmacists were more likely to disagree with a variety of existing drug classifications compared to those in Québec, which may be reflective of inherently greater control during sale in that province [20]. Also evident was a lack of support for drugs being unscheduled in Québec, which may suggest less confidence in the public’s ability to use the agents appropriately without pharmacy intervention. This study surveyed pharmacists in both provinces, revealing their preference for certain drugs to be reclassified. This suggests a potential shift in pharmacy practice towards greater independence in therapy initiation by pharmacists or more facilitated patient self-care.

As a window into how care might unfold today, Ontario and Québec pharmacists were surveyed to assess how they would approach two hypothetical heartburn cases. The selection of these two provinces was based on several factors, including their significance within the Canadian healthcare landscape (as the two biggest provinces in the country) and because of their distinct drug scheduling processes (i.e., the important differences in how heartburn medicines are regulated for sale). Ontario follows the scheduling recommendations provided by the National Association of Pharmacy Regulatory Authorities (NAPRA), which sets guidelines for scheduling medications (and subsequent conditions of their sale) across the country. Medications are categorized into four groups indicating the level of professional oversight necessary for their safe and effective use. Schedule I drugs necessitate a prescription for dispensing by pharmacists. Schedule II (“behind-the-counter” (BTC)) drugs do not mandate a prescription but require professional assistance at the point of sale, as they are not available for self-selection by the public. Schedule III (OTC) drugs can be obtained without a prescription but must be sold in a designated area under pharmacist supervision. Unscheduled drugs can be sold without professional oversight and are accessible in any retail establishment. On the other hand, Québec has its own (provincial) drug scheduling processes coordinated by the Office des professions du Québec, resulting in differences in scheduling decisions and conditions of sale compared to other provinces who schedule in reference to NAPRA. For example, Nexium (esomeprazole) 14-day therapy packs can be sold from the OTC aisles of pharmacies in Ontario, whereas it has been relegated to Schedule II (BTC) status in Québec. Additionally, ranitidine 75 mg can be sold from any retail outlet in Ontario but must be sold from pharmacies in Québec.

Of note, however, this study was not a quality assurance endeavor, where therapeutic decision-making is critiqued in relation to current standards. Rather, it was to describe what might take place in relation to case severity for a common ailment in community pharmacies and to possibly gauge how scheduling differences might dictate therapy choices. Two of the dynamics during case management—pharmacist confidence and consult duration—were also of interest.

## 2. Materials and Methods

### 2.1. Study Design and Population of Interest

This was a cross-sectional descriptive survey of hypothetical cases in heartburn management by practicing community pharmacists in Ontario (n = 11,658) and Québec (n = 7258) [21]. A total of 6507 initial survey invitations were sent to Ontario pharmacists. A comparable number for Québec pharmacists was not possible, as we could not estimate reach via social media. Based on the 2022 statistics from the NAPRA, 11,658 pharmacists were practicing in community pharmacies in Ontario and 7258 in Québec. Cochran’s formula, incorporating this population size and using a 5% margin of error and 95% confidence level, resulted in a minimum sample size of 377 respondents. A target sample size of 500 respondents was established, weighted to 60% of respondents from Ontario and 40% from Québec to proportionally reflect the number of practitioners in these provinces. Specifically, we aimed to recruit 308 participants from Ontario and 192 from Québec to hopefully ensure the sample size adequately represented the pharmacist population in each region.

Ontario and Québec were strategically chosen as the focal points of our study to encompass the broad spectrum of drug scheduling practices within Canada’s largest provinces. These provinces were selected due to their marked disparities in regulatory frameworks and decision-making procedures concerning drug scheduling. This deliberate selection facilitated the exploration of pharmacist perspectives and preferences amidst diverse provincial drug regulations.

### 2.2. Questionnaire Design

An English and French electronic survey instrument was constructed using the Qualtrics online platform (Qualtrics International Inc., Provo, UT, USA). This survey was pilot tested by 6 pharmacists (practicing in community and primary care), with modifications made based on their feedback. The final survey instrument consisted of 28 questions (Supplementary Materials). Question formats included item-specific scales, multiple-choice questions, and open text entry.

The pharmacists were asked to consider two cases at two levels of complexity and how they would approach management for both. The case content was created with guidance from hidden shopper studies covering a variety of topics [22,23,24,25], ones specific to dyspepsia [26,27], and a repertoire of 21 cases, which encompassed three distinct levels of heartburn severity and addressed optimal timelines for seeking medical care [28]. These considerations ensured that the selected cases were both clinically relevant and representative of scenarios commonly encountered in pharmacy practice.

#### 2.2.1. Case Wording for Lower Complexity Case (Case #1)

“A 26-year-old male complains of heartburn. He used to get it once or twice a month, but since changing jobs 2 months ago, he has been experiencing at least one episode every week. This burning in his chest generally occurs 30–60 min after he eats spicy and fatty foods, and sometimes leaves an acidic taste in the back of his mouth. He does not get a sore throat from this, it is never difficult to swallow, and there is no stomach pain. He has never seen a primary care provider or pharmacist about this issue and is hoping to find something so he can feel better at work. He is otherwise healthy, with no medical conditions or allergies and does not regularly take any medications”.

#### 2.2.2. Case Wording for Higher Complexity Case (Case #2)

“A 45-year-old male has experienced heartburn 4 times a week over the last 10 weeks. Each episode lasts about 40 min, then goes away. During that time, he gets an acidic taste in the back of his mouth, especially when he burps. Sometimes it can be caused by certain foods; other times there are no apparent reasons. But it is more common when he is lying down. He has never noticed any stomach pain from it. He has no other symptoms. He has never asked a primary care provider or pharmacist about this. On further inquiry, he reports that he has high blood pressure, mild diabetes, and osteoarthritis, all treated with medications. He is 30 pounds overweight. He has tried Gaviscon (alginic acid + magnesium carbonate) 2 chewable tablets after lunch, to relieve his symptoms in the past, but the relief is short-lived (return after about 45 min)”.

#### 2.2.3. Pharmacist Confidence and Consultation Duration

The pharmacists’ confidence in managing each case was measured on a seven-point Likert scale, with verbal anchors at each numerical point, ranging from 1 = not at all confident to 7 = completely confident.

Estimates of the time needed to assess the cases and make recommendations were based on ranges when dealing with first-time OTC medicine users across a variety of areas [29]. The therapeutic options available to the pharmacists were assembled via an iterative process by the research team, which included practicing pharmacists from both provinces. The pharmacists were asked to estimate the number of questions they get on the topic in a given month.

To garner the best reflection of actual practice behavior, the respondents were informed this was not an assessment of their clinical skill nor adherence to practice guidelines.

The final questionnaire was pre-tested and minor modifications were made. Reliability testing (assessing whether the pharmacists would vote for one therapeutic option initially and then provide a different answer if asked at another time) was not carried out nor was scale validity.

The survey was available in both English and French and was administered online by the Survey Research Centre (SRC) at the University of Waterloo.

### 2.3. Questionnaire Delivery

The steps undertaken to administer the survey have previously been described [20]. Email addresses of practicing community pharmacists in Ontario (who indicated a willingness to participate in practice research) were obtained from the provincial authority. All the pharmacists in this database were sent an email in advance to explain the nature of the project. This was followed by the main email with the link to the questionnaire. Two reminder emails were sent to non-respondents.

In Québec, an email address list was not available from the provincial authority, so an open (non-unique) survey URL link was used to advertise the survey more generally using social media platforms (Facebook and LinkedIn) and to collect responses from Québec pharmacists. Metadata were captured and reviewed to identify duplicate IP addresses. Completed questionnaires were adjudicated for authenticity, completeness, and target audience by the SRC. An incentive of a $5 Starbucks e-gift card was offered for each valid, completed document.

### 2.4. Data Analysis

The Statistical Package for the Social Sciences (SPSS; version 28, 2021) and Statistical Analysis System (SAS; version 9.4M6, 2018) were used to describe the data gathered in the online survey.

### 2.5. Ethics Approval

All identifying information (email and IP addresses) were removed from the final data file to ensure the confidentiality of the respondents. The University of Waterloo Research Ethics Board approved this study on ethical grounds in August 2022 (#44519).

## 3. Results

Data collection took place from 20 September to 19 October 2022. At survey close, 715 pharmacists had completed the survey, 462 from Ontario and 253 from Québec. A definitive response rate (as defined by the American Association for Public Opinion Research) for the entire study could not be calculated, as it was not possible to accurately identify the total number of Québec pharmacists who were made aware of the survey link on social media.

Sample re-weighting was not needed for the data, since the proportion of respondents from each province was aligned with the pre-specified weighting by province. The median time for questionnaire completion was 12 min.

### 3.1. Responder Characteristics

Responder demographics and primary places of employment have been described in a previous report [20]. There were proportionally more female respondents from Québec (58.2%) than Ontario (46.2%). Most were working 30 or more hours per week and had been a pharmacist for 1 to 10 years, most commonly as a staff pharmacist. At the pharmacy designated as their main location of employment, the majority were in large urban areas. Pharmacists practicing in independent pharmacies were more common in Ontario, with banner/franchise outlets being of note in both provinces. Steady (moderate pace) or busy with slow periods reflected the most common descriptions for what a typical day was like for the responding pharmacists.

### 3.2. Requests in a Month

The number of requests for advice that pharmacists receive per month for heartburn/GERD were estimated to be: none (Ontario = 26, 5.6 percent; Québec = 5, 2.0 percent), between one and five (Ontario = 141, 30.5 percent; Québec = 79, 31.2 percent), between 6 and 10 (Ontario = 165, 35.7 percent; Québec = 101, 39.9 percent), and greater than 10 (Ontario = 130, 28.2 percent; Québec = 68, 26.9 percent).

### 3.3. Confidence

Pharmacist confidence as measured by the seven-point Likert scale was similar across provinces and for both cases (Table 1). The proportions expressing moderately or very confident for case 1 were 60.6 percent and 58.5 percent (Ontario and Québec, respectively). For case 2, those selecting these same levels of confidence were 53.5 percent (Ontario) and 58.1 percent (Québec).

### 3.4. Duration of Consults

The responders estimated how long each consultation might take to properly assess (Table 2). About two-thirds (both provinces) determined that case 1 would need between three and five minutes. In an expected shift of greater involvement, the proportions of pharmacists choosing more than five minutes for case 2 was 24.9 percent (Ontario) and 32.4 percent (Québec).

### 3.5. Pharmacist Approach to Care

Details of which products the pharmacists would choose for the two cases appear in Table 3 and Table 4, respectively. A wide range of approaches were evident, including a number of pharmacists that opted for antacid therapy for case 1. The breakdown for Ontario was antacid (11.9 percent), solo H2RA (21.2 percent), combination H2RA + antacid (29.4 percent), nonRx PPI (22.3 percent), prescription H2RA (7.1 percent), prescription PPI (5.6 percent), other prescription (1.3 percent), and referral (1.5 percent) (Table 3). Regarding the choice between a single-entity H2RA versus a combination H2RA + antacid, Québec pharmacists chose the former by approximately a 2:1 ratio while those from Ontario had a higher likelihood for using a combination product.

In case 2, the breakdown for Québec was increase the Gaviscon dose (1.6 percent), add another antacid (4.7 percent), switch to OTC H2RA (22.1 percent), switch to combination H2RA + antacid (13.4 percent), switch to nonRx PPI (24.9 percent), switch to prescription H2RA (2.4 percent), switch to prescription PPI (22.5 percent), switch to another prescription (0.8 percent), or referral (7.5 percent) (Table 4).

For case 1, 1.5 percent of the Ontario pharmacists opted to refer to medical care (Table 3). In case 2, this percentage rose to 9.5 percent (Table 4), with relatively similar proportions in Québec.

If the patients with the circumstances of case 1 had returned to the pharmacy because their symptoms had not resolved with therapy as recommended, 20.2 percent and 24.7 percent of Québec and Ontario pharmacists, respectively, would tell the patient to seek medical care (Table 3). Referral rates more than doubled for case 2 (Table 4), if a patient returned within a week.

### 3.6. Impact of Drug Scheduling on Approach to Care

When asked whether the scheduling status of the various heartburn drugs was a factor in determining which therapeutic agent(s) they went on to recommend, 71.0 percent and 62.0 percent of Ontario and Québec pharmacists, respectively, agreed that this was indeed a factor.

## 4. Discussion

This report outlines the therapeutic decision-making of pharmacists in two Canadian provinces. As such, it is one of the few that describes a clinical approach to a condition in the practice environment, in this case, heartburn.

The main findings are that pharmacists would be confident in actually dealing with two such situations, with over half being at least moderately so, which did not see a precipitous drop when the case rose in complexity. This is a common symptom seen in community pharmacies, and repetition likely plays a role in that finding.

There was a significant level of diversity in approaches for each case and with both provinces. For example, about one in five pharmacists in Ontario either chose a single-entity H2RA, one combined with an antacid, or a nonRx PPI for case 1 (Table 3). For case 2, similar proportions were seen in Québec between the choices of an OTC H2RA, a nonRx PPI, or a prescribed PPI (Table 4). The most common option chosen by Ontario pharmacists for case 1 was a combination product of H2RA + antacid, while a single-entity H2RA was most common with their Québec counterparts (Table 3).

For case 2, both groups opted most commonly for an OTC H2RA either alone or in combination with an antacid (Ontario = 96 + 81 and Québec = 56 + 34) (Table 4). This was followed in frequency by a nonRx PPI (Ontario = 134 and Québec = 63). Choosing what was available to them via pharmacist prescribing (of either an H2RA or PPI) occurred in 67 and 63 instances for Ontario and Québec, respectively. It should be noted that linking first therapeutic choices to specific follow-up choices seven days later was not possible. It is therefore possible that a pharmacist could have opted for a prescribed PPI at initial contact, then kept with it upon the patient’s return. We did, however, capture whether changes were made in a general sense (Table 3 and Table 4). Percentage-wise, the pharmacists in Québec engaged in their right to prescribe a PPI (57 of 253) more often than those in Ontario (42 of 462). It appeared that the patients returning with symptoms seven days later (case 1) were most commonly switched to another entity, rather than having the patient continue with the agent originally initiated. With case 2, although switching to another agent had a strong response in Ontario, the prevailing approach in both provinces was to refer patients.

Other findings were that approximately two-thirds of the pharmacists in both provinces estimated that case 1 would need between 3 and 5 min to assess (Table 2). As one would expect, the pharmacists felt more time would be needed for the case with greater complexity. Just under 10 pharmacists in each province felt that each case could be handled in less than two minutes.

As one would also expect, referral rates for returning patients significantly rose for both cases. Initial referrals for case 1 were decidedly low. If patients returned in 7 days with unresolved symptoms, approximately one in five Québec pharmacists (and one in four Ontario pharmacists) would refer at that juncture. The referral rates for case 2 were initially 9.5 percent (Ontario) and 7.5 percent (Québec), then rose to 42.4 percent (Ontario) and 55.7 percent (Québec) upon patients returning to the pharmacy in 7 days without resolution. It would appear that the pharmacists are cautious in their approach to care management, although there was no pre-determined percentage that was deemed to be the appropriate level for referral. The referral patterns could also have an inverse relationship with pharmacist confidence: as confidence drops, referral rates could rise (as clinical skills are surpassed). This was not formally assessed, but given the feedback on confidence, it might make sense that the pharmacists felt reasonably confident in knowing when medical care was needed, rather than a sense of feeling overwhelmed.

The other literature that documents what pharmacists recommend for heartburn cases is limited. Krishnan and Schaefer examined the outcomes of pharmacist advice-giving but not the agents chosen [7]. Aradottir and Kinnear outlined a treatment algorithm for dyspepsia management in community pharmacies but that did not extend to the choices made [30].

However, in Australia, pharmacists responded to a disguised shopper with NSAID-induced reflux [15]. Across 223 pharmacy visits, recommendations for treatment included an H2RA (57 percent), an antacid (19 percent), and a PPI (18 percent). Referral to a doctor was made in 63 percent of the visits. NSAID-induced reflux would likely be considered more serious than case 2 by most healthcare providers, which could explain the higher referral rates seen in this report. In another notable exception, a report from the United Arab Emirates outlined what pharmacists recommended for a simulated patient with GERD [31]. Using data collected from visits to 150 community pharmacies, 93 encounters resulted in a PPI being recommended, followed by 58 suggesting an antacid, and one for an H2RA, sometimes in combination with each other (as in, 17 opted for a PPI + antacid). However, as a separate entity, 73 chose a PPI. The authors noted there was a significant difference in approaches between the pharmacists in the two cities under study, with no speculation as to why that may have occurred.

Referral rates in general are more commonly reported [7,13,27]. Researchers in Belgium investigated the nature of GI symptoms that people intended to treat with OTC medicines, the prevalence of any alarm symptoms, adherence to medical referral advice, and the benefit seen with product use. Almost 600 patients were recruited in 63 community pharmacies. At least one alarm symptom was present in 22.4 percent of patients. Although 21 percent of the clients were referred to medical care, only 51.7 percent did so [8]. In Sweden, only one patient in five who were advised to see a physician actually did so [14]. Over 80 percent claimed to have followed the pharmacist’s advice, with two of three saying they felt better after doing so.

This all represents rather new and unique information for the Canadian landscape. The regulation of drug scheduling and point of sale is a pivotal aspect of public health policy, exhibiting notable variations across jurisdictions. The regulation of nonRx drugs in Canada involves both federal and provincial authorities. Health Canada approves health products for sale based on safety, quality, and efficacy, at times enabling OTC status. Provincial governments primarily dictate drug scheduling and conditions of sale through regulations associated with their Pharmacy Acts. Most provinces (including Ontario) align with the National Drug Schedules established by a national body (NAPRA), but Québec maintains an independent process, leading to discrepancies in drug scheduling and subsequent conditions of sale.

These variances hold significant implications for healthcare delivery, patient access, and regulatory enforcement. Ontario’s decentralized framework offers flexibility to adapt to regional needs, while Québec’s centralized approach aims to streamline pharmaceutical procurement and distribution. However, Québec’s independent scheduling process, marked by sluggishness and lack of transparency, results in delays in access to innovative self-care medicines. Despite Québec’s Schedule II default policy that was to facilitate market entry, lengthy decision-making processes hinder timely access to newly available products, leading to disparities in ingredient access compared to the rest of Canada.

The insights regarding potential efficiency gains in provincial healthcare systems stem from a 2017 report by The Conference Board of Canada titled “The Impact of Switching Prescription Medications to Over-the-Counter” [32]. This report employed an economic model to evaluate the economic consequences of transitioning PPIs from prescription to OTC status for the management of GERD. Through the model’s analysis, various dimensions of economic impact, including time savings, productivity enhancements, and cost transfer implications resulting from the switch, were assessed. These dimensions encompassed aspects such as healthcare system utilization, medication acquisition, the financial burden associated with treatment, and labor productivity. The derived results provided insights into the potential financial gains associated with such a policy change, with specific emphasis placed on the Ontario and Québec contexts. In Ontario, where 36% of PPIs are prescribed, the projected efficiency gains for the provincial government (as a result of fewer primary care visits and PPI prescriptions) were estimated at $66.9 million, while Québec stood to benefit from gains totalling $41.2 million [32]. It is important to highlight that these efficiency gains would be moderated with pharmacists prescribing PPIs, as provincial drug plans would remain responsible for covering these agents, albeit with varying extents of coverage.

### Limitations

There was a strong volunteer component to the results, in that the pharmacists had to agree to participate in the process.

The subjects in both provinces were not randomly selected from any sampling frame. It should be noted that more female pharmacists from Québec responded, which could bias the data. Furthermore, the Québec pharmacists entered the process by seeing an advertisement for it on social media.

Of critical importance is that the results reflect what pharmacists say they will do, not necessarily what they actually might do in practice.

## 5. Conclusions

Our study highlights distinct approaches to managing heartburn among pharmacists in Ontario and Québec, revealing variations both within and across these populous Canadian provinces. These findings offer valuable insights into the diversity of therapeutic choices within this domain, offering fertile ground for stimulating discourse regarding optimal care strategies. Notably, the pharmacists demonstrated a pronounced inclination towards referring the more complex case to medical care. Furthermore, our findings underscore pharmacists’ notable level of confidence in approaching heartburn cases, reflecting their expertise and preparedness in managing this common ailment. In addition, given the recent shift of PPIs from prescription to nonRx status in Canada, our study offers valuable perspectives into how this regulatory change may influence pharmacists’ approaches to heartburn therapy in two distinct provinces. This highlights the significance of drug scheduling on patient care and prompts consideration of optimal therapeutic and regulatory approaches.

## Figures and Tables

**Table 1:** Confidence in managing cases.

Confidence Level	Heartburn Case 1		Heartburn Case 2	
	Ontario n (%)	Québec n (%)	Ontario n (%)	Québec n (%)
Not at all confident	1 (0.2%)	1 (0.4%)	1 (0.2%)	1 (0.4%)
Not very confident	13 (2.8%)	9 (3.6%)	16 (3.5%)	12 (4.7%)
Slightly confident	38 (8.2%)	28 (11.1%)	54 (11.7%)	34 (13.4%)
Somewhat confident	76 (16.5%)	24 (9.5%)	91 (19.7%)	41 (16.2%)
Moderately confident	122 (26.4%)	66 (26.1%)	144 (31.2%)	106 (41.9%)
Very confident	158 (34.2%)	82 (32.4%)	108 (23.4%)	41 (16.2%)
Completely confident	54 (11.7%)	43 (17%)	47 (10.2%)	18 (7.1%)

Footnote: n = 462 in Ontario; n = 253 in Québec.

**Table 2:** Time likely needed for cases.

Estimated Time	Heartburn Case 1		Heartburn Case 2	
	Ontario n (%)	Québec n (%)	Ontario n (%)	Québec n (%)
Under 2 min	9 (2.0%)	8 (3.2%)	7 (1.5%)	6 (2.4%)
Around 2 to 3 min	99 (21.4%)	36 (14.2%)	61 (15.2%)	29 (11.5%)
Around 3 to 4 min	153 (33.1%)	85 (33.6%)	132 (28.6%)	58 (22.9%)
Around 4 to 5 min	134 (29.0%)	85 (33.6%)	147 (31.8%)	78 (30.8%)
More than 5 min	67 (14.5%)	39 (15.4%)	115 (24.9%)	82 (32.4%)

Footnote: n = 462 in Ontario; n = 253 in Québec.

**Table 3:** Treatment of heartburn—case 1 (Ontario n = 462, Québec n = 253 respondents).

	Ontario n (%)	Québec n (%)
**What therapy would you initiate for this patient (if any)?**		
Antacid (e.g., calcium carbonate)	55 (11.9%)	42 (16.6%)
Non-prescription H2RA (e.g., Zantac regular strength)	96 (20.8%)	84 (33.2%)
Non-prescription H2RA + antacid (e.g., Pepcid Complete)	136 (29.4%)	44 (17.4%)
Non-prescription PPI (e.g., Nexium 24HR)	103 (22.3%)	39 (15.4%)
Prescription H2RA (e.g., nizatidine)	33 (7.1%)	10 (4.0%)
Prescription PPI (e.g., pantoprazole)	26 (5.6%)	27 (10.7%)
Other prescription agent (e.g., sucralfate, metoclopramide, domperidone)	6 (1.3%)	6 (2.4%)
None of the above—refer patient to a primary care provider	7 (1.5%)	1 (0.4%)
**If the patient returns to you in 7 days and states the symptoms have not resolved, persisted at the same intensity, but no additional alarm symptoms have developed, what would be your next course of action?**		
Continue with your initial therapy for a few more weeks	81 (17.5%)	38 (15.0%)
Switch to a different prescription or nonprescription agent	267 (57.8%)	164 (64.8%)
None of the above—refer patient to a primary care provider	114 (24.7%)	51 (20.2%)

Footnote: H2RA: histamine-2 receptor antagonist; PPI: proton pump inhibitor; n = 462 in Ontario; n = 253 in Québec.

**Table 4:** Treatment of heartburn—case 2 (Ontario n = 462, Québec n = 253 respondents).

	Ontario n (%)	Québec n (%)
**What therapy would you initiate for this patient (if any)?**		
Increase the dose of the current regimen (Gaviscon) to 4–5 times a day	9 (1.9%)	4 (1.6%)
Add another antacid (e.g., calcium carbonate) to the current regimen	22 (4.8%)	12 (4.7%)
Switch to a nonprescription H2RA (e.g., Zantac regular strength)	96 (20.8%)	56 (22.1%)
Switch to a nonprescription H2RA + antacid (e.g., Pepcid Complete)	81 (17.5%)	34 (13.4%)
Switch to a nonprescription PPI (e.g., Nexium 24HR)	134 (29.0%)	63 (24.9%)
Switch to a prescription H2RA (e.g., nizatidine)	25 (5.4%)	6 (2.4%)
Switch to a prescription PPI (e.g., pantoprazole)	42 (9.1%)	57 (22.5%)
Switch to another prescription agent (e.g., sucralfate, metoclopramide, domperidone)	9 (1.9%)	2 (0.8%)
None of the above—refer patient to a primary care provider	44 (9.5%)	19 (7.5%)
**If the patient returns to you in 7 days and states the symptoms have not resolved, persisted at the same intensity, but no additional alarm symptoms have developed, what would be your next course of action?**		
Continue with your initial therapy for a few more weeks	85 (18.4%)	42 (16.6%)
Switch to another prescription or nonprescription agent	181 (39.2%)	70 (27.7%)
None of the above—refer patient to a primary care provider	196 (42.4%)	141 (55.7%)

Footnote: H2RA: histamine-2 receptor antagonist; PPI: proton pump inhibitor.

## Data Availability

SPSS data files can be made available to those who request it.

## Supplementary Materials

The following supporting information can be downloaded at: https://www.mdpi.com/article/10.3390/pharmacy12030081/s1, Screening Questions.
