# Supplementary material for: Treatment of Heartburn: A Survey of Ontario and Québec Community Pharmacists
Source: Pharmacy (Basel). 2024 May 22;12(3):81. doi: 10.3390/pharmacy12030081 (PMC11130813; doi:10.3390/pharmacy12030081)
Supplement: Supplementary file 1 [file pharmacy-12-00081-s001.zip › pharmacy-2958034-supplementary.pdf]

Screening Questions

If left blank, the following message should pop up, "This is an essential question that will determine your eligibility."

Section 0: Pre-Screening Question (to determine eligibility for completion of survey)

S1: Which province(s) are you currently practicing community pharmacy in?

01 Ontario
02 Quebec
03 None of the above

If S1 = 03, go to NE

Not Eligible (NE)

Thank you for your interest in our project. Unfortunately, we are only inviting pharmacists who are currently practicing in the community pharmacies in Ontario or Quebec. We thank you kindly for your interest in our study and hope you have a great day.

Programming instructions
• Help Box to appear at the top of every page

HELP BOX

If you require technical help with the survey, please contact the Survey Research Centre at srcccinb@uwaterloo.ca.

For any questions about the study itself, contact the investigators of this study:

Principal Investigator:
Dr. Nardine Nakhla, Pharm D, School of Pharmacy, University of Waterloo. Phone: 1-519-888-4567 ext. 21340, Email: nardine.nakhla@uwaterloo.ca

Co-Investigators:
Dr. Sherilyn Houle, PhD, School of Pharmacy, University of Waterloo. Email: sherilyn.houle@uwaterloo.ca
Dr. Jeff Taylor, College of Pharmacy and Nutrition, University of Saskatchewan, Email: jeff.taylor@usask.ca
Dr. Sadaf Faisal, PhD, School of Pharmacy, University of Waterloo. Email: sadaf.faisal@uwaterloo.ca
Dr. Francis Richard, Pharm D, MScPhm, BCMTMS, Université de Montréal. Email: francis.richard@umontreal.ca

This study has been reviewed by, and received ethics clearance, through a University of Waterloo Research Ethics Board (REB #44519). If you have questions for the Board, contact the Office of Research Ethics, at 519-888-4567 ext. 36005 or reb@uwaterloo.ca.

[End of help box]

Survey Questions – 3 sections

Programming instructions
• Question order static
• Answer choices randomized as indicated

Programmer: Error prompt to appear on all the following questions if response not provided, "There is an unanswered question on this page. Would you like to continue? If so, please click "Next" again. Otherwise, please answer the question above."

Section 1: Demographic Questions

Q1: On average**,** how **many hours per week** do you work in community pharmacy?

a. None **(go to NE2 message)**
b. 1-14 hours/week
c. 15-29 hours/week
d. 30-39 hours/week
e. 40 hours or more

Q2: Please indicate the **number of years** you have been practising pharmacy?

a. Less than 1 year
b. 1-5 years
c. 6-10 years
d. 11-15 years
e. 16-20 years
f. More than 20 years

Q3: What **gender** do you identify with?

a. Male
b. Female
c. Transgender
d. Non-binary
e. Prefer to self-describe: _____________
f. Prefer not to answer

Programmer: Display NE2 message if option a "None" is ticked: "Thank you, unfortunately you will not be able continue the survey as we are looking for community pharmacists who are currently working at least 1 hour/week in a community pharmacy. We thank you kindly for your interest in our study and hope you have a great day.**"**

Q4: To ensure we are capturing your answers correctly, please select all the fruits from the following list:

a. Strawberry
b. Horse
c. Apple
d. Squirrel
e. Banana

Programmer: Display Q5-Q8 on one page.

Many of us work in several locations. Please answer the following questions in relation to the community pharmacy that you primarily work in.

Q5: Where is the pharmacy where you primarily work located?

a. Large urban population centre (population of 100,000 or more)
b. Medium population centre (population between 30,000 - 99,999)
c. Small population centre (population between 1,000 - 29,999)
d. Rural area (population of less than 1,000)

Q6: What does a **typical day** in the dispensary of that pharmacy look like?

a. Slow
b. Slow with busy periods
c. Steady moderate pace
d. Busy with slow periods
e. Busy

Q7: What **type of community pharmacy** is it?

a. Independent
b. Chain associated with Supermarket (like Loblaws, Sobeys)
c. Mass merchant/discount retailer (like Costco, Walmart)
d. Banner/Franchise store (like Shoppers Drug Mart, Pharma Save, PharmaChoice, Jean Coutu, Uniprix))
e. Other, please specify: _______________

Q8: Which of the following would **best describe your role at this community pharmacy**? Please select all that apply.

a. Pharmacy owner
b. Pharmacy manager/Chief Pharmacist
c. Staff pharmacist
d. Locum/relief pharmacist
e. Other, please specify: _______________

Section 2: Minor Ailments of Interest

Q9: On average, how many requests for advice do you receive **per month** for the following ailments?

	**None**	**Between 1-5**	**Between 6-10**	**More than 10**
Vulvovaginal Candidiasis				
Heartburn/GERD				
Allergic Rhinitis				

Section 3: Drug Scheduling

PROGRAMMER, PLEASE PROVIDE LOGIC TO DETERMINE IF PARTICIPANT IS FROM ONTARIO OR QUEBEC THEN DISPLAY THE CORRESPONDING TABLE.

Q10: The table below lists the current scheduling status of common drugs used for **heartburn, allergic rhinitis, and vulvovaginal candidiasis**. We are interested in your opinion about the status of each drug in the province where you work.

If you need a refresher on the definitions of the various schedules [click here].

If participant is from **Ontario**, the following table will appear: **PROGRAMMER, DO NOT SHOW IF S1 = 02**

**Drug Name**	**Current Status**	**What should it be in your opinion?**			
Terconazole (Taro-terconazole) for intravaginal use	Schedule I	☐Schedule I	☐ Schedule II	☐ Schedule III	☐ Unscheduled
Clotrimazole (Canestenâ) for intravaginal use	Schedule III	☐Schedule I	☐ Schedule II	☐ Schedule III	☐ Unscheduled
Miconazole (Monistatâ) for intravaginal use	Schedule III	☐ Schedule I	☐ Schedule II	☐ Schedule III	☐ Unscheduled
Fluconazole 150 mg (Diflucanâ One), when sold as 1 capsule package size	Schedule III	☐ Schedule I	☐ Schedule II	☐ Schedule III	☐ Unscheduled
Fluconazole 150 mg + clotrimazole external cream (CanesOralâ Combi-Pak)	Schedule III	☐ Schedule I	☐ Schedule II	☐ Schedule III	☐ Unscheduled
Esomeprazole 20 mg (Nexium 24HRâ), when sold as 14-day treatment package	Schedule III	☐ Schedule I	☐ Schedule II	☐ Schedule III	☐ Unscheduled
Omeprazole 20 mg, when sold as 14-day treatment package	Schedule II	☐ Schedule I	☐ Schedule II	☐ Schedule III	☐ Unscheduled

Famotidine 40 mg (Teva-Famotidine)	Schedule I	☐ Schedule I	☐ Schedule II	☐ Schedule III	☐ Unscheduled
Ranitidine 75 mg, when sold in package sizes containing ≤4500 mg	Unscheduled	☐ Schedule I	☐ Schedule II	☐ Schedule III	☐ Unscheduled
Rupatadine 10 mg (Rupallâ)	Schedule I	☐ Schedule I	☐ Schedule II	☐ Schedule III	☐ Unscheduled
Diphenhydramine 25 mg (Benadrylâ)	Schedule III	☐ Schedule I	☐ Schedule II	☐ Schedule III	☐ Unscheduled
Loratadine 5 mg + Pseudoephedrine 120 mg (Claritinâ Allergy + Sinus)	Schedule III	☐ Schedule I	☐ Schedule II	☐ Schedule III	☐ Unscheduled
Fluticasone propionate 50 mcg intranasal spray (Flonaseâ Allergy Relief), when sold as package sizes containing **NO MORE THAN** 360 metered sprays, for use in ages 18 and up	Schedule III	☐ Schedule I	☐ Schedule II	☐ Schedule III	☐ Unscheduled
Mometasone 50 mcg intranasal spray (Nasonexâ), for use in ages 12 and up	Schedule I	☐ Schedule I	☐ Schedule II	☐ Schedule III	☐ Unscheduled
Triamcinolone 55 mcg intranasal spray (Nasacortâ Allergy 24HRâ), when sold as package sizes containing **NO MORE THAN** 120 metered sprays, for use in ages 12 and up	Schedule III	☐ Schedule I	☐ Schedule II	☐ Schedule III	☐ Unscheduled

If participant is from **Quebec**, the following table will appear: **PROGRAMMER, DO NOT SHOW IF S1 = 01**

Drug Name	Current Status	What should it be in your opinion?			
Terconazole (Taro-terconazole) for intravaginal use	Schedule I	☐Schedule I	☐ Schedule II	☐ Schedule III	☐ Unscheduled
Clotrimazole (Canestenâ) for intravaginal use	Schedule III	☐Schedule I	☐ Schedule II	☐ Schedule III	☐ Unscheduled
Miconazole (Monistatâ) for intravaginal use	Schedule III	☐ Schedule I	☐ Schedule II	☐ Schedule III	☐ Unscheduled
Fluconazole 150 mg (Diflucanâ One), when sold as 1 capsule package size	Schedule II	☐ Schedule I	☐ Schedule II	☐ Schedule III	☐ Unscheduled
Fluconazole 150 mg + clotrimazole external cream (CanesOralâ Combi-Pak)	Schedule II	☐ Schedule I	☐ Schedule II	☐ Schedule III	☐ Unscheduled
Esomeprazole 20 mg (Nexium 24HRâ), when sold as 14-day treatment package	Schedule II	☐ Schedule I	☐ Schedule II	☐ Schedule III	☐ Unscheduled
Omeprazole 20 mg, when sold as 14-day treatment package	Schedule II	☐ Schedule I	☐ Schedule II	☐ Schedule III	☐ Unscheduled
Famotidine 40 mg tablet (Teva-Famotidine)	Schedule I	☐ Schedule I	☐ Schedule II	☐ Schedule III	☐ Unscheduled

Ranitidine 75 mg	Schedule III	☐ Schedule I	☐ Schedule II	☐ Schedule III	☐ Unscheduled
Rupatadine 10 mg (Rupall[ª])	Schedule I	☐ Schedule I	☐ Schedule II	☐ Schedule III	☐ Unscheduled
Diphenhydramine 25 mg (Benadryl[ª])	Schedule III	☐ Schedule I	☐ Schedule II	☐ Schedule III	☐ Unscheduled
Loratadine 5 mg + Pseudoephedrine 120 mg (Claritin[ª] Allergy + Sinus), when sold as package sizes containing **MORE THAN** 10 tablets	Schedule II	☐ Schedule I	☐ Schedule II	☐ Schedule III	☐ Unscheduled
Fluticasone propionate 50 mcg intranasal spray (Flonase[ª] Allergy Relief), for use in ages 18 and up	Schedule II	☐ Schedule I	☐ Schedule II	☐ Schedule III	☐ Unscheduled
Mometasone 50 mcg intranasal spray (Mosaspray[ª]), for use in ages 12 and up	Schedule II	☐ Schedule I	☐ Schedule II	☐ Schedule III	☐ Unscheduled
Triamcinolone 55 mcg intranasal spray (Nasacort[ª] Allergy 24HR[ª]), for use in ages 12 and up	Schedule II	☐ Schedule I	☐ Schedule II	☐ Schedule III	☐Unscheduled

Q11: To ensure we are capturing your answers correctly, please select all the animals from the following list:

a. Dog
b. Blue
c. Tiger
d. Monkey
e. Orange

Patient Cases

This next section of the survey asks about four cases concerning three minor ailments. Please answer based on how you would address each case if the patient visited your pharmacy *today* (predicated on your *current* allowable scope of practice).

Case 1:

A 30-year-old healthy female approaches you complaining of vaginal and vulvar itching and a white, curdy vaginal discharge. She has been experiencing these symptoms for 3 days. She denies an odour to the discharge, is not sexually active and has recently tested negative for sexually transmitted infections. She has no other symptoms. The vaginal pH was just self-measured using an over-the-counter pH indicator test and came back negative (pH is within normal limits). She experienced similar symptoms last year and the doctor recommended visiting the pharmacy to pick up a vaginal cream, which cleared up all symptoms. Her only other medication is Alesse-28. She has no medical conditions and no product preferences. While she does not have third party drug coverage, cost is not an issue, as she will gladly pay for any agent that will effectively resolve her bothersome symptoms.

Q12a: What therapy would you initiate for this patient (if any)?

a. Fluconazole oral tablet 150mg x 1 (e.g., Diflucanâ One)
b. Clotrimazole intravaginal agent (e.g., Canestenâ)
c. Miconazole intravaginal agent (e.g., Monistatâ)
d. Terconazole intravaginal agent (e.g., Taro-Terconazole)
e. Combination product containing an intravaginal antifungal + external antifungal agent (e.g., Monistatâ combination pack)
f. Combination product containing an oral antifungal + external antifungal agent (e.g., CanesOralâ Combi-Pak)
g. None of the above; Refer patient to a primary care provider **(skip to Q13)**

Q 12b: How would you initiate therapy for the medication(s) selected above?

a. Prescribe a Schedule I agent
b. Prescribe a Schedule II or III agent
c. Recommend purchasing a Schedule II or III agent
d. Contact the prescriber and then prescribe a Schedule I agent

Q13: How long would you anticipate this consultation to take on average?

a. Under 2 minutes
b. Around 2 to 3 minutes
c. Around 3 to 4 minutes
d. Around 4 to 5 minutes
e. More than 5 minutes

Q14: In your province, is the scheduling status of the drugs to manage this condition a factor in determining which therapeutic agent(s) you recommend/prescribe?

a. Yes
b. No

IF you would like to explain further, please do:

Case 2:

A 26-year-old male complains of heartburn. He used to get it once or twice a month, but since changing jobs 2 months ago, he has been experiencing at least one episode every week. This burning in his chest generally occurs 30-60 minutes after he eats spicy and fatty foods, and sometimes leaves an acidic taste in the back of his mouth. He does not get a sore throat from this, it is never difficult to swallow, and there is no stomach pain. He has never seen a primary care provider or pharmacist about this issue and is hoping to find something so he can feel better at work. He is otherwise healthy, with no medical conditions or allergies and does not regularly take any medications.

Q15: How CONFIDENT ARE YOU in your ability to handle this case?

Not at all confident	Not very confident	Slightly confident	Somewhat confident	Moderately confident	Very confident	Completely confident
1	2	3	4	5	6	7

Q16a: What therapy would you initiate for this patient (if any)?

a. Antacid (e.g., calcium carbonate)
b. Non-prescription H_2 receptor antagonist (e.g., Zantac[â] regular strength)
c. Non-prescription H_2 receptor antagonist + antacid (e.g., Pepcid[â] Complete)
d. Non-prescription proton pump inhibitor (e.g., Nexium 24HR[â])
e. Prescription H_2 receptor antagonist (e.g., nizatidine)
f. Prescription proton pump inhibitor (e.g., pantoprazole)
g. Other prescription agent (e.g., sucralfate, metoclopramide, domperidone)
h. None of the above; Refer patient to a primary care provider **(skip to Q17)**

Q 16b: How would you initiate therapy for the medication(s) selected above?

a. Prescribe a Schedule I agent
b. Prescribe a Schedule II or III agent
c. Recommend purchasing a Schedule II or III agent
d. Contact the prescriber and then prescribe a Schedule I agent

Q17: How long would you anticipate this consultation to take on average?

a. Under 2 minutes
b. Around 2 to 3 minutes
c. Around 3 to 4 minutes
d. Around 4 to 5 minutes
e. More than 5 minutes

Q18a: If the patient returns to you in 7 days and states the symptoms have not resolved, persisted at the same intensity, but no additional alarm symptoms (e.g., epigastric pain or vomiting) have developed, what would be your next course of action?

a. Continue with your initial therapy for a few more weeks **(skip to Q19)**
b. Switch to a different prescription or non- prescription agent
c. None of the above; Refer patient to a primary care provider **(skip to Q19)**

Q18b: Please select which of the following agent you would recommend:

a. Antacid (e.g., calcium carbonate)
b. Non-prescription H_2 receptor antagonist (e.g., Zantac® regular strength)
c. Non-prescription H_2 receptor antagonist + antacid (e.g., Pepcidâ Complete)
d. Non-prescription proton pump inhibitor (e.g., Nexium 24HRâ)
e. Prescription H_2 receptor antagonist (e.g., nizatidine)
f. Prescription proton pump inhibitor (e.g., pantoprazole)
g. Other prescription agent (e.g., sucralfate, metoclopramide, domperidone)
h. Other, please specify: _______________

Q19: In your province, is the scheduling status of the drugs to manage this condition a factor in determining which therapeutic agent(s) you recommend/prescribe?

a. Yes
b. No

IF you would like to explain further, please do:

Case 3:

A 45-year-old male has experienced heartburn 4 times a week over the last 10 weeks. Each episode lasts about 40 minutes, then goes away. During that time, he gets an acidic taste in the back of his mouth, especially when he burps. Sometimes it can be caused by certain foods; other times there are no apparent reasons. But, it is more common when he is lying down. He has never noticed any stomach pain from it. He has no other symptoms. He has never asked a primary care provider or pharmacist about this. On further inquiry, he reports that he has high blood pressure, mild diabetes, and osteoarthritis, all treated with medications. He is 30 pounds overweight. He has tried Gavisconâ (alginic acid + magnesium carbonate) 2 chewable tablets after lunch, to relieve his symptoms in the past, but the relief is short-lived (return after about 45 minutes).

Q20: How CONFIDENT ARE YOU in your ability to handle this case?

Not at all confident	Not very confident	Slightly confident	Somewhat confident	Moderately confident	Very confident	Completely confident
1	2	3	4	5	6	7

Q21a: What therapy would you initiate for this patient (if any)?

a. Increase the dose of the current regimen (Gaviscon) to 4-5 times a day
b. Add another antacid (e.g., calcium carbonate) to the current regimen
c. Switch to a non-prescription H_2 receptor antagonist (e.g., Zantacâ regular strength)
d. Switch to a non-prescription H_2 receptor antagonist + antacid (e.g., Pepcid® Complete)
e. Switch to a non-prescription proton pump inhibitor (e.g., Nexium 24HR®)
f. Switch to a prescription H_2 receptor antagonist (e.g., nizatidine)
g. Switch to a prescription proton pump inhibitor (e.g., pantoprazole)
h. Switch to another prescription agent (e.g., sucralfate, metoclopramide, domperidone)
i. None of the above; refer patient to a primary care provider **(skip to Q22)**

Q 21b: How would you initiate therapy for the medication(s) selected above?

a. Prescribe a Schedule I agent
b. Prescribe a Schedule II or III agent
c. Recommend purchasing a Schedule II or III agent
d. Contact the prescriber and then prescribe a Schedule I agent

Q22: How long would you anticipate this consultation to take on average?

a. Under 2 minutes
b. Around 2 to 3 minutes
c. Around 3 to 4 minutes
d. Around 4 to 5 minutes
e. More than 5 minutes

Q23a: If the patient returns to you in 7 days and states the symptoms have not resolved, persisted at the same intensity, but no additional alarm symptoms (e.g., epigastric pain or vomiting) have developed, what would be your next course of action?

a. Continue with your initial therapy for a few more weeks **(skip to Q24)**
b. Switch to another prescription or non- prescription agent
c. None of the above; Refer to a primary care provider **(skip to Q24)**

Q23b: Please select which of the following agent you would recommend:

a. Non-prescription H_2 receptor antagonist (e.g., Zantacâ regular strength)
b. Non-prescription H_2 receptor antagonist + antacid (e.g., Pepcidâ Complete)
c. Non-prescription proton pump inhibitor (e.g., Nexium 24HR®)

d. Prescription H_2 receptor antagonist (e.g., nizatidine)
e. Prescription proton pump inhibitor (e.g., pantoprazole)
f. Other prescription agent (e.g., sucralfate, metoclopramide, domperidone)
g. Other, please specify: _______________

Q24: In your province, is the scheduling status of the drugs to manage this condition a factor in determining which therapeutic agent(s) you recommend/prescribe?

a. Yes
b. No

IF you would like to explain further, please do:

Case 4:

A 28-year-old female has a very runny nose and is sneezing frequently during the day. Her eyes and nose are always itchy and bothersome. Occasionally, her nose can get congested (blocked) too. She notes that her symptoms get worse in the summer months and on windy days. On more troublesome days, getting her chores done is more difficult. The symptoms can keep her up at night. She has NO other symptoms. This has occurred since she was 10 years old. She has never seen a physician, nurse practitioner, or pharmacist for this issue. Her neighbour recommended she take an oral decongestant + analgesic for this, which helps minimally, but does not remember its name.

Q25a: What therapy would you initiate for this patient (if any)?

a. Continue with oral decongestant + analgesic combination therapy
b. Non-prescription oral first-generation antihistamine (e.g., diphenhydramine)
c. Non-prescription oral second-generation antihistamine (e.g., loratadine)
d. Non-prescription intranasal corticosteroid spray (e.g., triamcinolone)
e. Non-prescription oral second-generation antihistamine + oral decongestant (e.g., loratadine + pseudoephedrine)
f. Prescription intranasal corticosteroid spray (e.g., ciclesonide)
g. Prescription oral second-generation antihistamine (e.g., rupatadine)
h. None of the above; refer patient to a primary care provider **(skip to Q26)**

Q 25b: How would you initiate therapy for the medication(s) selected above?

a. Prescribe a Schedule I agent
b. Prescribe a Schedule II or III agent
c. Recommend purchasing a Schedule II or III agent
d. Contact the prescriber and then prescribe a Schedule I agent

Q26: How long would you anticipate this consultation to take on average?

a. Under 2 minutes
b. Around 2 to 3 minutes
c. Around 3 to 4 minutes
d. Around 4 to 5 minutes
e. More than 5 minutes

Q27a: If this patient returns in 14 days and complains of continuing symptoms, at the same intensity, but NO alarm symptoms (e.g., shortness of breath, wheezing or facial pain), what would be your next course of action?

a. Continue with your initial therapy for a few more weeks **(skip to Q28)**
b. Switch to another prescription or non-prescription option **(Skip to Q 27b)**
c. Add on another prescription or non-prescription option **(Skip to Q27c)**
d. None of the above; refer patient to a primary care provider **(skip to Q28)**

Q27b: Please select which of the following option you would switch to:

a. Non-prescription oral first-generation antihistamine (e.g., diphenhydramine)
b. Non-prescription oral second-generation antihistamine (e.g., loratadine)
c. Non-prescription intranasal corticosteroid spray (e.g., triamcinolone)
d. Non-prescription oral second-generation antihistamine + oral decongestant (e.g., loratadine + pseudoephedrine)
e. Prescription intranasal corticosteroid spray (e.g., ciclesonide)
f. Prescription oral second-generation antihistamine (e.g., rupatadine)
g. Other, please specify: _______________

Q27c: Please select which of the following options you would add:

a. Non-prescription oral first-generation antihistamine (e.g., diphenhydramine)
b. Non-prescription oral second-generation antihistamine (e.g., loratadine)
c. Non-prescription intranasal corticosteroid spray (e.g., triamcinolone)
d. Non-prescription oral second-generation antihistamine + oral decongestant (e.g., loratadine + pseudoephedrine)
e. Prescription intranasal corticosteroid spray (e.g., ciclesonide)
f. Prescription oral second-generation antihistamine (e.g., rupatadine)
g. Other, please specify: _______________

Q28: In your province, is the scheduling status of the drugs to manage this condition a factor in determining which therapeutic agent(s) you recommend/prescribe?

a. Yes
b. No

IF you would like to explain further, please do:

Compensation
This section asks for your consent to contact you to provide compensation for your participation in this survey. As a reminder, survey responses will be de-identified. Your personal information will be stored separately and will only be used to provide compensation.

Q29: Would you like to receive compensation in the form of a $5 Starbucks electronic gift card for participating in this survey?

a. Yes
b. No **(go to CLOSING)**

Programmer, clicking yes to the above will connect participants to a separate contact form.

Contact Form

Thank you, a $5 Starbucks electronic gift card will be sent to the email you provide within the next 7-10 days. Depending on your email settings, the gift card notification may go into your Junk Mail so please check there if you have not received the gift card notification within 7 to 10 days. If you do not receive your gift card notification after 10 days, please email uwsrc@uwaterloo.ca.

First Name
Last Name
Email Address
Telephone Number

Please note that the researchers and their institutions reserve the right to refuse incentive payment for any survey completions that the researchers and their institutions deem fraudulent or only partially completed.

CLOSING

Thank You!
You have reached the end of the survey. We would like to thank you very much for your time, we know you are busy and have many other commitments!

If you wish to obtain a summary report of the study, please contact:

Principal Investigator:
Dr. Nardine Nakhla, Pharm D, School of Pharmacy, University of Waterloo. Phone: 1-519-888-4567 ext. 21340, Email: nardine.nakhla@uwaterloo.ca

Co-Investigators:
Dr. Sherilyn Houle, PhD, School of Pharmacy, University of Waterloo. Email: sherilyn.houle@uwaterloo.ca
Dr. Jeff Taylor, College of Pharmacy and Nutrition, University of Saskatchewan. Email: jeff.taylor@usask.ca
Dr. Sadaf Faisal, PhD, School of Pharmacy, University of Waterloo. Email: sadaf.faisal@uwaterloo.ca
Dr. Francis Richard, Pharm D, MScPhm, BCMTMS, Université de Montréal. Email: francis.richard@umontreal.ca
